# Tianhuang formula regulates adipocyte mitochondrial function by AMPK/MICU1 pathway in HFD/STZ-induced T2DM mice

**DOI:** 10.1186/s12906-023-04009-5

**Published:** 2023-06-19

**Authors:** Duosheng Luo, Yaru Zhao, Zhaoyan Fang, Yating Zhao, Yi Han, Jingyu Piao, Xianglu Rong, Jiao Guo

**Affiliations:** Guangdong Metabolic Diseases Research Center of Integrated Chinese and Western Medicine; Key Laboratory of Glucolipid Metabolic Disorder, Ministry of Education of China; Institute of Chinese Medicine, Guangdong Pharmaceutical University, Guangzhou Higher Education Mega Center; Guangdong TCM Key Laboratory for Metabolic Diseases, 280 Wai Huan Dong Road, Guangzhou, 510006 China

**Keywords:** Type 2 diabetes mellitus, Adipose tissue, Mitochondrial function, AMPK, MICU1

## Abstract

**Background:**

Tianhuang formula (THF) is a Chinese medicine prescription that is patented and clinically approved, and has been shown to improve energy metabolism, but the underlying mechanism remains poorly understood. The purpose of this study is to clarify the potential mechanisms of THF in the treatment of type 2 diabetes mellitus (T2DM).

**Methods:**

A murine model of T2DM was induced by high-fat diet (HFD) feeding combined with low-dose streptozocin (STZ) injections, and the diabetic mice were treated with THF by gavaging for consecutive 10 weeks. Fasting blood glucose (FBG), serum insulin, blood lipid, mitochondrial Ca^2+^ (mCa^2+^) levels and mitochondrial membrane potential (MMP), as well as ATP production were analyzed. The target genes and proteins expression of visceral adipose tissue (Vat) was tested by RT-PCR and western blot, respectively. The underlying mechanism of the regulating energy metabolism effect of THF was further explored in the insulin resistance model of 3T3-L1 adipocytes cultured with dexamethasone (DXM).

**Results:**

THF restored impaired glucose tolerance and insulin resistance in diabetic mice. Serum levels of lipids were significantly decreased, as well as fasting blood glucose and insulin in THF-treated mice. THF regulated _m_Ca^2+^ uptake, increased MMP and ATP content in VAT. THF increased the mRNA and protein expression of AMPK, phosphorylated AMPK (p-AMPK), MICU1, sirtuin1 (SIRT1) and peroxisome proliferator-activated receptor-γ coactivator-1α (PGC-1α). THF could increase the _m_Ca^2+^ level of 3T3-L1 adipocytes and regulate mitochondrial function. The protein expression of AMPK, p-AMPK, _m_Ca^2+^ uniporter (MCU) and MICU1 decreased upon adding AMPK inhibitor compound C to 3T3-L1 adipocytes and the protein expression of MCU and MICU1 decreased upon adding the MCU inhibitor ruthenium red.

**Conclusions:**

These results demonstrated that THF ameliorated glucose and lipid metabolism disorders in T2DM mice through the improvement of AMPK/MICU1 pathway-dependent mitochondrial function in adipose tissue.

**Supplementary Information:**

The online version contains supplementary material available at 10.1186/s12906-023-04009-5.

## Highlights

• AMPK-MCU/MICU1 mediated _m_Ca^2+^ uptake in adipose tissue played an important role in insulin resistance

• THF restored impaired glucose tolerance and insulin resistance in diabetic mice induced by HFD/STZ

• THF improved the mitochondrial function of  in T2DM mice via AMPK-MICU1 pathway

**Supplementary Information:**

The online version contains supplementary material available at 10.1186/s12906-023-04009-5.

## Background

Diabetes mellitus (DM) is a chronic and lifelong illness with growing incidence globally and is characterized by inadequate insulin secretion [1]. Mitochondria are known as cellular ‘power plants’ [2], their dysfunction plays a pivotal role in the processes of diabetes [3], and can lead to a variety of medical problems including neurodegenerative diseases, cardiovascular diseases, cancer, and metabolic disorders. The mitochondrial energy metabolism disorder will be further aggravated during the development of diabetes. They have an influence on each other through common or different mechanisms, and even form a vicious circle. Mitochondrial dysfunction, caused by _m_Ca^2+^ homeostasis imbalance under a high glucose environment, may be one of the important pathological mechanisms of diabetes and its complications [4]. The _m_Ca^2+^ transport is a complex and strictly controlled process. Mitochondrial calcium uniporter (MCU) complex has been identified as a major channel located on the inner membrane to regulate Ca^2+^ transport into mitochondria [5]. Silencing MCU in cultured cells or in vivo in mouse liver severely abrogates mitochondrial Ca^2+^ uptake [6]. And there is growing evidence that MICU1 is a gatekeeper of MCU-mediated _m_Ca^2+^ uptake that is essential to prevent _m_Ca^2+^ overload and associated stress [7]. Therefore, MCU/MICU1 may play a fundamental role in mediating _m_Ca^2+^ homeostasis in T2DM, and may represent a novel therapeutic target for T2DM.

Insulin resistance (IR) is the principal pathogenesis of T2DM as an initiating risk factor. The main target organs for insulin action are the liver, the muscle and the adipose tissue. Adipose tissue is a dynamic endocrine organ and nutrient sensor that tightly regulates energy supply. Such as insulin can maintain glucose homeostasis by stimulating glucose uptake in adipose tissue and reducing hepatic gluconeogenesis. The recent trend in research on the mechanism of adipose tissue leading to T2DM mainly focuses on specific adipokines, inflammation and metabolism, that is, adipose tissue dysfunction, which promotes inflammation, hyperlipidemia and IR, and finally results in T2DM [8, 9]. However, there are only very limited reports on how _m_Ca^2+^ homeostasis in adipose tissue affects T2DM. Mitochondria play a fundamental role in maintaining the balance of energy homeostasis in metabolic tissues, including adipose tissues, and participate in the regulation of energy metabolism homeostasis through fatty acid oxidation [10], oxidative phosphorylation (OXPHOS) [11] and _m_Ca^2+^ uptake [12]. The AMPK is a sensor of cellular energy status that regulates cellular and whole-body energy balance. During cell mitosis, AMPK mediates the phosphorylation on the 57th serine of MCU to promote the large amount of Ca^2+^ to enter the mitochondria and stimulate ATP produced to change the state of insufficient cell energy [13]. However, the current research evidence is still insufficient to prove that AMPK, MCU and MICU1 are involved in the regulation of _m_Ca^2+^ uptake in diabetic adipose tissue.

The Traditional Chinese Medicine Fufang Zhenshu Tiaozhi Formula (FTZ), was developed based on *Prof.* Jiao Guo's 30 years of clinical experience, and has now been developed into a hospital preparation. Studies showed that FTZ has been well-documented with significant clinical curative effects for hyperglycemia and hyperlipidemia [14, 15]. FTZ has the effect of controlling blood sugar and keeping patients' blood sugar stable for a long time [16], reducing the serum levels of TC, TG, LDL-C and Non-HDL-C in patients [17]. Because of its excellent cost-effective properties, FTZ capsules have been covered by health insurance in the Guangdong Province of China. In clinical and experimental studies over the past few years, a simpler but equivalent formula originated from the FTZ has formed, named Tianhuang formula (THF), which is a patented and clinically approved Chinese medicinal prescription composed of *Radix Notoginseng(Panax notoginseng (Burkill) F.H.Chen ex C.Y.Wu & K.M.Feng)* and *Rhizoma Coptidis (Coptis chinensis Franch)*. Previous studies have shown THF could regulate lipid metabolism disorders through the gut microbiota-TβMCA-FXR metabolism axis [18], and improve the insulin resistance and glucose intolerance of obese rats induced by high fat and sugar diets [19]. In addition, *Radix Notoginseng* and *Rhizoma Coptidis* are both traditional herbal drugs with hundreds of years of usage. A large number of studies have shown that *Radix Notoginseng and Rhizoma Coptidis* have anti-hyperglycemic activity [20–22]. In conclusion, THF has the potential to improve glucose metabolism, but its mechanism has not been fully elucidated.

This study demonstrated that the AMPK-MICU1 pathway mediated _m_Ca^2+^ uptake, affected mitochondrial function, and caused an imbalance in energy metabolism, further leading to glucose and lipid metabolism disorders in Vat of STZ-induced T2DM mice. THF attenuated T2DM by regulating _m_Ca^2+^ uptake in Vat which mechanism may be mediated through the AMPK-MICU1 pathway. These data addressed key gaps in our understanding of the AMPK-MICU1 and shed light on the role MICU1 plays in T2DM.

## Methods

### Herbal materials

Herbs in THF ( *Radix Notoginseng* and *Rhizoma Coptidis*) were provided by Zhixin Chinese Herbal Medicine Co., Ltd. (Guangzhou, China. S). *Prof.* Jiao Guo, Guangdong Pharmaceutical University authenticated the plant material using the Pharmacopeia of the People's Republic of China identification key (ISBN 2020, volume I). The production batch numbers were 210,401 and 210,501. Plant names have been checked with http://www.theplantlist.org.

### Preparation and chemical constituents of THF

THF was prepared as follows [19], powdered *Radix Notoginseng* (400 g) and *Rhizoma Coptidis* (400 g) were separately extracted triply with 70% ethanol at 80 °C under reflux, each time for 2 h. The extract solution was concentrated in a rotary evaporator to remove ethanol, and then dissolved in water and purified using D101 macro-porous resin (Lanxiao, Xi’an). The resulting purified extract was dried in a vacuum at 60 °C. The quantitative profiling of THF was performed on a U3000 HPLC with a DAD detector (Dionex, USA). The chromatography separation was carried out using a Kromasil C_18_ column (4.5 × 250 mm, 5 μm in particle size) according to the Pharmacopoeia of the People’s Republic of China (2020), and data were recorded and analyzed on the Chromeleon Console workstation. Finally, the content of eight active components in THF, namely, Ginsenoside Rg1, Ginsenoside Rb1, Ginsenoside Rd, Ginsenoside Re, Notoginsenoside R1, Berberine, Coptisine, and Palmatine, were quantified.

### Animals and management ethics protocol

All the animal experiments were approved by the Animal Ethical Committee of Guangdong Pharmaceutical University (SPF2017310). Specific pathogen free (SPF) male C57BL/6 J Narl mice 3–4 weeks of age were purchased from Guangdong Medical Laboratory Animal Center. All mice were housed in a temperature-controlled room at 24 °C ± 2 °C, with a humidity of 60%-70%, and 12 h of light and darkness alternated, standard solid food and water were provided during the experiment.

### Induction of hyperglycemia in experimental animals

Mice were divided into two groups, normal and diabetic groups. To induce diabetes, mice were fed HFD (60% fat, 20% protein, 20% carbohydrate, Research Diets, D12492) for 4 weeks [23]. Then, the mice were fasted for 6 h followed by the administration of intraperitoneal (ip) injection of 40 mg/kg STZ for 4 consecutive days based on the previous study by *Gilbert ER* et al. [24]. The STZ (Sigma, St. Louis, MO, USA) was dissolved in citrate buffer (0.05 M, pH4.5), which was freshly prepared before use. Blood glucose level was checked using an Accu-Chek blood glucometer (Roche Diagnostics, Basel, Switzerland) every 72 h. Stable hyperglycemia was established if the fasting blood glucose (FBG) was ≥ 11.1 mmol/L in the tested animals after 1 week from the STZ injection. Normal mice received i.p. injection of citrate buffer.

Mice were allocated randomly after 1 week from diabetes induction into three groups (n = 8) whereas the normal mice were allocated randomly after 1 week from ip injection of citrate buffer (n = 8) and treated daily as follows for 6 weeks. Control group: normal mice received oral normal saline only. Model group: animals were fed HFD and received STZ. High-dose THF (THF-H) group: HFD/STZ diabetic mice were treated with THF (120 mg/kg/day). Low-dose THF (THF-L) group: HFD/STZ diabetic mice were treated with THF (60 mg/kg/day) [19]. Metformin (MET) group: HFD/STZ diabetic mice were administered MET (250 mg/kg).

The body weight and food intake of the mice were assessed once per week. The fat mass was detected in the last week of the experiment using the Minispec LF90 Body Composition Analyzer (Bruker). For the oral glucose tolerance test (OGTT), mice were given oral glucose (2 g/kg) after fasting for 6 h to measure their glucose tolerance, and the blood glucose levels at different time points were detected using the Accu-Chek blood glucometer immediately after the initial injection of glucose. The areas under the curves (AUC) of the glucose level over time were calculated to evaluate the glucose tolerance ability of the mice [25].

After all the experiments, all mice were euthanized with 200 mg/kg pentobarbital sodium through intraperitoneal injection, blood samples were collected from the mice’s orbital venous plexus and transferred into a collection tube, which were then centrifuged at 3500 rpm for 30 min at 4 °C, serum samples were prepared and kept at -80 °C [19]. The TC, TG, APN and insulin levels in serum were measured using commercial kits according to the manufacturers' introductions. Adipose tissue was collected and stored at -80 °C until subsequent biochemical analyses and was fixed in 10% buffered formalin for histopathological examination [25]. Animal bodies were taken care of by the Animal Ethical Committee of Guangdong Pharmaceutical University.

### Histopathological screening

Adipose tissue specimens were fixed in 10% formalin for 24 h, dehydrated using gradual ethanol concentrations, and embedded in paraffin. Then, the paraffin- embedded specimens were sectioned into 5 μm thick sections and stained with hematoxylin and eosin (H&E)[25]. The adipose tissue (1 mm × 1 mm × 1 mm) was placed in a 2.5% glutaraldehyde fixative solution, fixed overnight at 4 °C, dehydrated, and observed under an electron microscope. Slides were examined under a light microscope (magnification: × 200, Eclipse E200-LED, Nikon, Tokyo, Japan).

### Metabolic rate measurements

The mice’s metabolism was evaluated by the Comprehensive Lab Animal Monitoring System (CLAMS, Columbus Instruments) according to the manufacturer's instructions. The mice were placed in individual cages and acclimated to the monitoring system for 24 h. The metabolic rate was evaluated by their carbon dioxide production (VCO_2_), oxygen consumption (VO_2_) and heat production over the next 24 h, which were analyzed with the CLAX Research software package (CLAX Research; CLAMS, Columbus Instruments)[25].

### Determination of mitochondrial Ca^2+^, MMP and ATP content in E-Wat and 3T3-L1 adipocyte

Fresh visceral adipose tissue (100 mg) was washed with PBS, then cutted into pieces and added 10 times the amount of pre-cooled mitochondrial separation reagent A, homogenized at low temperature, centrifuged to obtain the precipitate. Then the separated mitochondria in epididymal white adipose tissue (E-Wat) and 3T3-L1 adipocytes were added to the mitochondrial storage solution to detect the contents of mCa^2+^, MMP and ATP (Beyotime Biotechnology Co., Ltd., Shanghai, China) according to the mitochondrial membrane potential detection kit instructions.

### Cell culture and induction of adipocyte differentiation

3T3-L1 preadipocytes were cultured and differentiated into adipocytes by using a previously reported method [26]. Briefly, 3T3-L1 preadipocytes were cultured in DMEM containing 10% bovine calf serum at 37 °C in a 5% CO_2_ incubator. To induce differentiation, 2-day post-confluent preadipocytes were incubated for 2 days in a differentiation medium containing 10% FBS, 0.5 mM IBMX, 1 μM dexamethasone, and l μg/mL insulin. The medium was then changed to DMEM containing 10% FBS and 1 μg/mL insulin, and cells were cultured for another 2 days. Then cells were incubated in DMEM supplemented with 10% FBS for 2 more days.

### RNA extraction and quantitative real-time PCR

Total RNA was extracted using the Trizol reagent (Takara, Dalian, China). The RNA was transcribed into cDNA using a reverse transcription kit (Takara, Dalian, China) according to the manufacturer's instructions, and quantitative real-time PCR was performed using a Light Cycler 480 real-time PCR system (Roche, Switzerland). β- actin was used as an internal control to normalize expression values. The sequences of the PCR primers were listed in Supporting Table 1.

### Protein extraction and Western blotting analysis

Western blotting analysis was performed according to the protocol as previously reported [16]. Protein was extracted by RIPA lysis buffer (P1003B, Beyotime, China) and protein concentration was detected by the BCA kit (P0011, Beyotime, China). The primary antibodies were incubated at 4°Covernight, and the information of the brand and item number of the primary antibody were listed in Supporting Table 2. After incubating proper secondary antibodies, the protein bands were visualized by enhanced chemiluminescence (ECL) kit (Bio-Rad, CA, USA) and quantified by Image Pro Plus software.

### Statistical analysis

Data were presented as means ± standard error of mean (SEM). Data sets that involved more than two groups were assessed by one-way ANOVA followed by Newman-Keuls post hoc tests. *p* < 0.05 was considered statistically significant. GraphPad Prism 6.0 software (GraphPad, CA, USA) was used for statistical analysis and graphics.

## Result

### Preparation and quantitative profiling of THF

**Fig. 1:**
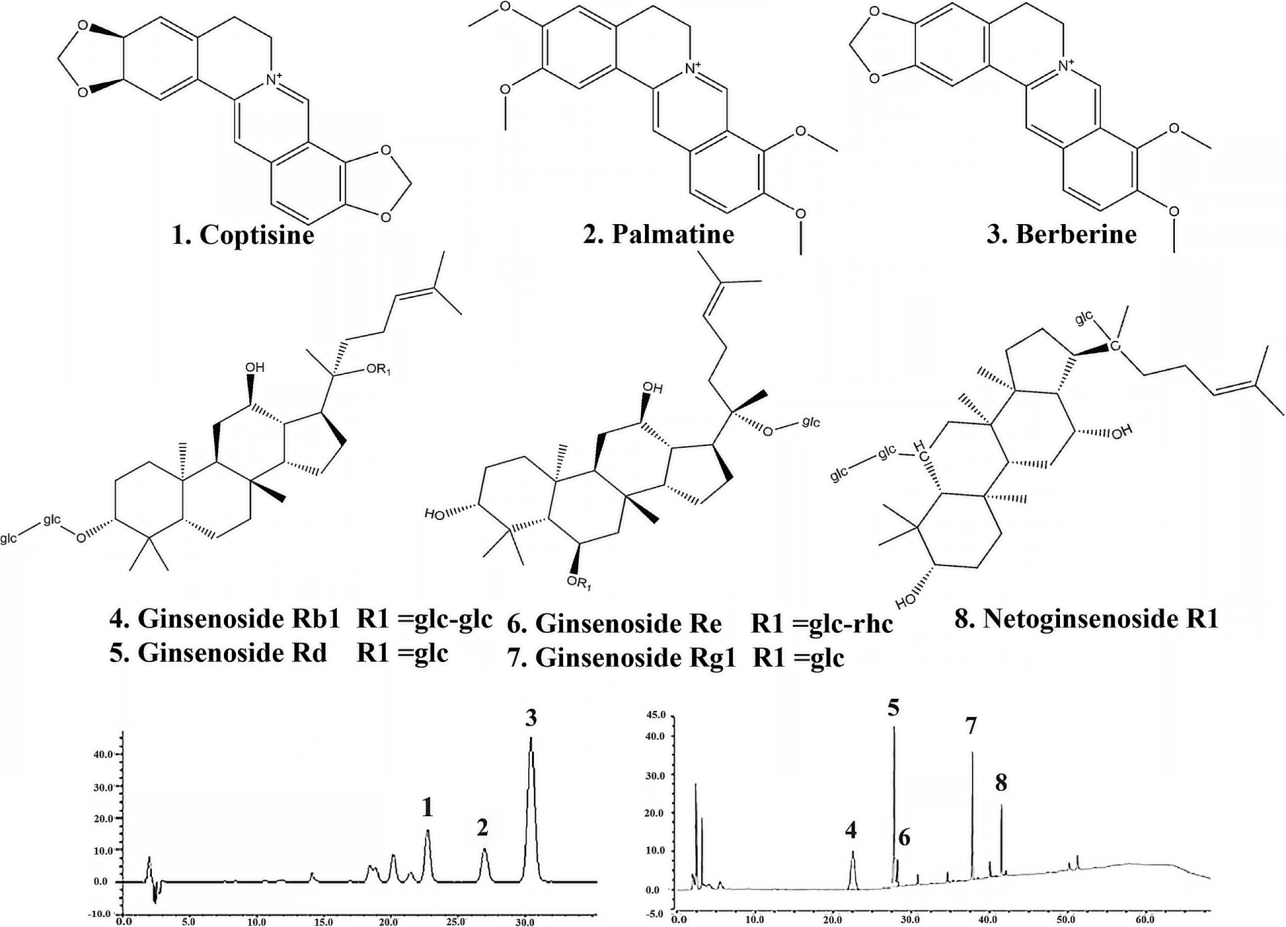
Preparation and quantitative profiling of Tianhuang formula. HPLC fingerprint chromatograms of the extracts of the reference standards (1) Ginsenoside Rg1 (CAS:), (2) Berberine (CAS:2086–83-1), (3) Palmatine (CAS:3486–67-7), (4) Ginsenoside Rb1 (CAS:41,753–43-9), (5) Coptisine (CAS: 3486–66-6), (6) Panax notoginseng saponin R1 (CAS:80,418–24-2), (7) Ginsenoside Re (CAS:52,286–59-6), (8) Ginsenoside Rd (CAS:52,705–93-8)

The results of the HPLC fingerprint chromatogram showed that THF was made of Ginsenoside Rg1 (23.82%), Berberine (17.01%), Palmatine (5.11%), Ginsenoside Rb1 (4.58%), Coptisine (4.45%), Panax notoginseng saponin R1 (2.05%), Ginsenoside Re (1.03%), Ginsenoside Rd (0.97%) and some other unidentified components (41.98%) (Fig. 1).

### THF improved the glucose and lipid metabolism in T2DM mice

**Fig. 2:**
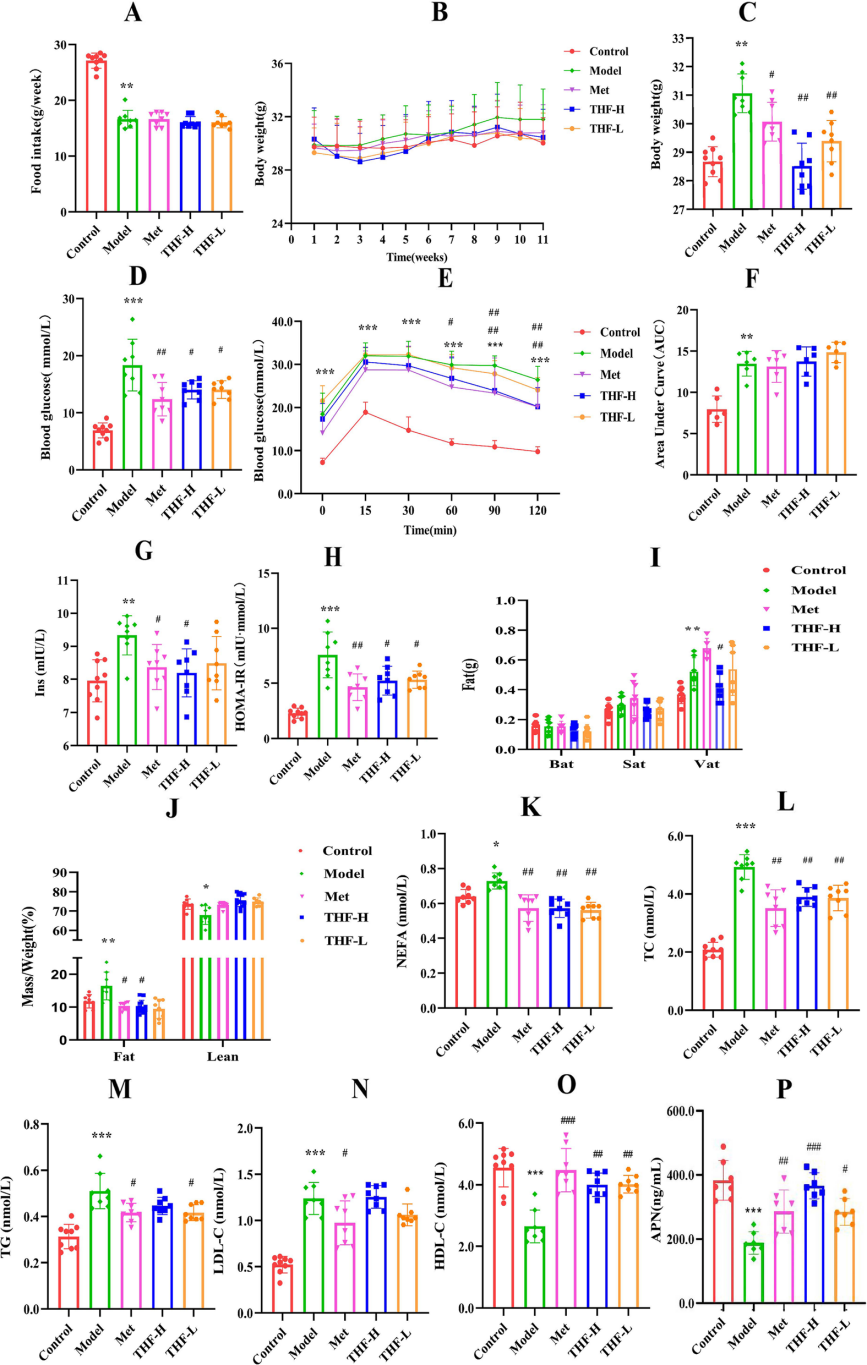
Comparison of indices during the construction of the HFD/STZ-induced T2DM mice and the treatment effects of THF. **A** Food intake. **B**-**C** Body weight. **D** Blood glucose. **E**–**F** OGTT and AUC analysis of OGTT. **G** Serum insulin. **H** HOMA-IR index. **I** Bat, Sat and Vat weigh. **J** MRI analysis of lean and fat mass. **K** Nonestesterified fatty acid (NEFA). **L** Triglyceride. **M** Total cholesterol. **N** Low-density lipoprotein. **O** High-density lipoprotein. **P** Adiponectin (APN). Notes:^*^P < 0.05, ^**^P < 0.01, ^***^P < 0.001 *vs* Control, ^#^*P* < 0.05, ^##^*P* < 0.01, ^###^*P* < 0.001 vs Model. All the data were presented as the means ± SEMs (*n* = 6–8)

Notably, there was no difference in food intake (Fig. 2A) between the model group and THF group. Body weights as shown in Fig. 2B-C, which were lower in THF group mice relative to model group mice. Compared with the control group, blood glucose levels were significantly increased in the model group and decreased in the THF treatment group (Fig. 2D). The AUC of OGTT results showed significant deterioration in glucose tolerance in the model group. Except for the control group, the glucose levels began to rise after oral glucose, peaking within 15 min, before gradually returning to the initial level. At 60 min, there was a significant difference between the model group and the Met group. After 90 min, glucose levels were significantly reduced in THF-H group (Fig. 2E and F). Compared with the control group, the fasting serum insulin level and IR index of the model group increased significantly, which was alleviated by metformin or THF treatment (Fig. 2G and H).

There were no differences in the weight of brown adipose and subcutaneous adipose among the groups. However, compared with the control group, the weight of E-Wat increased significantly in the model group and decreased significantly in the THF-H group (Fig. 2I). The body fat and the Fat/Weight ratio increased significantly and the Lean/Weight ratio showed a downward trend in the model group, which was significantly adjusted by metformin or THF treatment. It was speculated that the effect of THF on improving glucose and lipid metabolism disorders may be related to E-Wat dysfunction (Fig. 2J).

Furthermore, the nonestesterified fatty acid (NEFA), TC, LDL-C and TG levels in the serum of the model group mice significantly increased compared to the control group, while metformin or THF decreased the serum levels of NEFA, TC, LDL-C and TG (Fig. 2K-N). Serum levels of HDL-C and APN were decreased in HFD/STZ induced mice, while resumed after metformin or THF treatment (Fig. 2O), and the improvement of APN in the THF-H group was significantly better than in the other groups (Fig. 2P).

### THF improved the mitochondrial function of VAT in T2DM mice

**Fig. 3:**
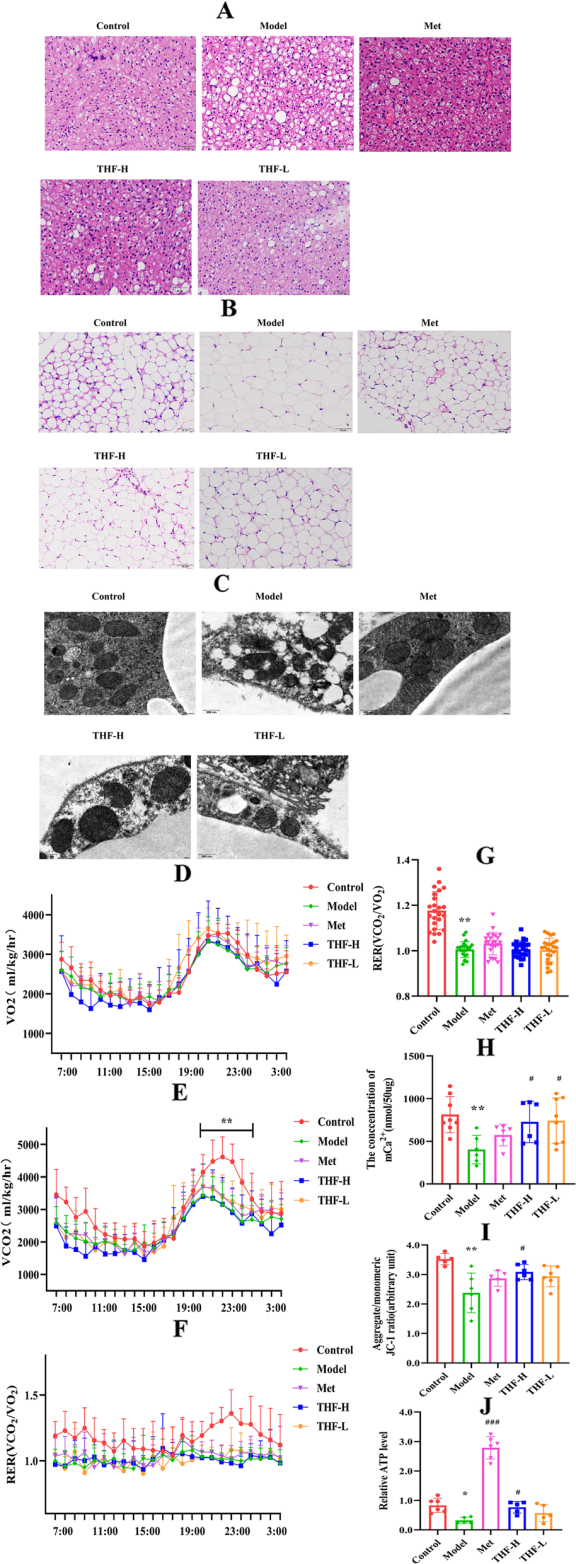
THF-treated mice exhibited improved mitochondrial structure. **A** Representative images of H&E-stained Bat of T2DM mice. **B** Representative images of H&E-stained epididymal E-Wat of T2DM mice. **C** Representative images of electron microscopy of E-Wat. **D** Oxygen consumption (VO_2_). **E** Carbon dioxide (VCO_2_). **F**-**G** VCO_2_/VO_2_ and AUC analysis of VCO_2_/VO_2_. **H** Mitochondrial Ca^2+^ levels of E-Wat. **I** Mitochondrial membrane potential MMP levels of E-Wat. **J** Relative ATP levels of E-Wat. Notes:^*^P < 0.05, ^**^P < 0.01, ^***^P < 0.001 *vs* Control, ^#^*P* < 0.05, ^##^*P* < 0.01, ^###^*P* < 0.001 vs Model. All the data were presented as the means ± SEMs (n = 6–8). Scale: 50 μm

Histologically, H&E revealed that the brown adipocytes (Bat) were hypertrophy and larger lipid droplets were formed in the adipocytes, and the number of lipid droplets was significantly increased in the model group (Fig. 3A). The visceral adipose cells were full of lipid droplets challenged with the HFD/STZ-induced (Fig. 3B). Observation of E-Wat by electron microscope showed that adipocyte was unclear, the mitochondria were swollen, and the cristae were difficult to distinguish in the model group, whereas THF and metformin treatment were capable to repress those histopathological signatures (Fig. 3C).

We used CLAMS to determine energy expenditure, and found that THF increased oxygen consumption (VO_2_) and carbon dioxide production (VCO_2_) during a 12-h cycle of light and dark. Systemic energy expenditure was significantly increased in THF and Met groups (Fig. 3D-G).

The mitochondrial function of Vat was further tested to investigate whether improvement of glucose and lipid metabolism following THF-treated was related to _m_Ca^2+^ homeostasis in adipose tissue. The _m_Ca^2+^ level of E-Wat was significantly reduced in the model group, which was increased significantly by THF treatment. And the levels of MMP and ATP decreased in T2DM mice. However, both metformin and THF-H treatments showed significant improvements (Fig. 3H-J). The mitochondrial function of Wat was impaired in a high-glucose environment and THF attenuated it.

### THF improved the mitochondrial function of E-Wat in T2DM mice through the AMPK-MICU1 pathway

**Fig. 4:**
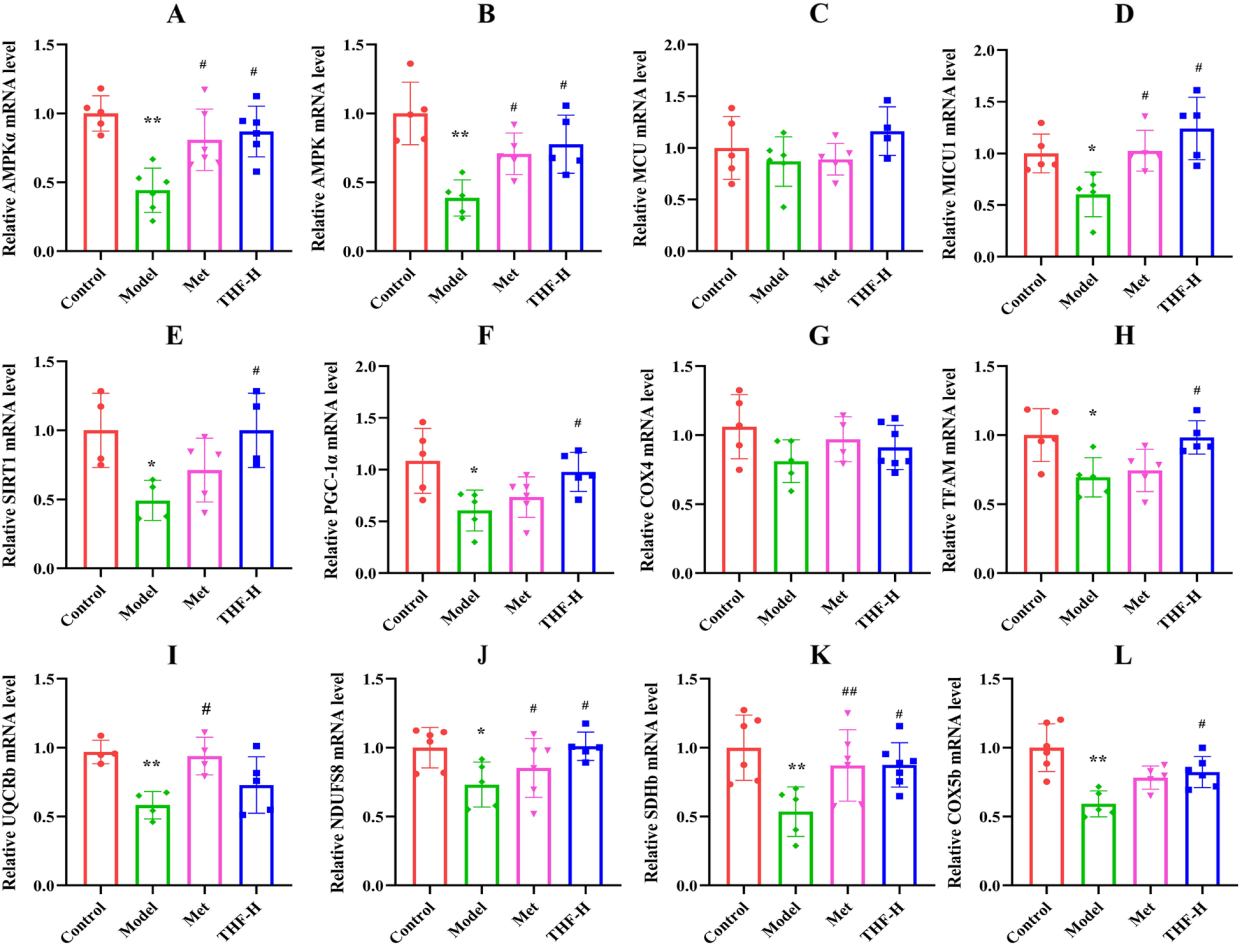
THF-H promoted mitochondrial energy metabolism-related mRNA expression in epididymal white adipose tissue of HFD/STZ-induced T2DM mice. **A**-**B** mRNA expression of AMPKα and AMPK. **C**-**D** mRNA expression of MCU and MICU1. **E**–**F** mRNA expression of SIRT1 and PGC-1α. **G**-**L** mRNA expression of COX4, TFAM, UQCRb, NDUFS8, SDHb and COX5b. Notes:^*^P < 0.05, ^**^P < 0.01, ^***^P < 0.001 *vs* Control, ^#^*P* < 0.05, ^##^*P* < 0.01, ^###^*P* < 0.001 vs Model. All the data were presented as the means ± SEMs (*n* = 4–6)

The results of the above experiments showed that THF-H improved T2D better than THF-L, indicating that THF improved T2D in a dose-dependent manner, to further investigate whether the protective mechanism of THF on mitochondrial function was linked to the AMPK-MICU1 pathway, we analyzed the mRNA expression levels of energy metabolism-related genes (AMPK, AMPKα, SIRT1, PGC-1α), MCU and MICU1, which decreased in the E-Wat of each group. Notably, MCU mRNA expression levels were indistinguishable between the control group and the model group. THF-H restored the mRNA expression of AMPK, AMPKα, p-AMPK, SIRT1, PGC-1α, and MICU1 (Fig. 4A-F). In addition, HFD/STZ-induced also significantly decreased mitochondrial function-specific genes expression of COX4, TFAM, UQCRb, NDUFS8, SDHb and COX5b, which further were upregulated by THF-H (Fig. 4G-L).

**Fig. 5:**
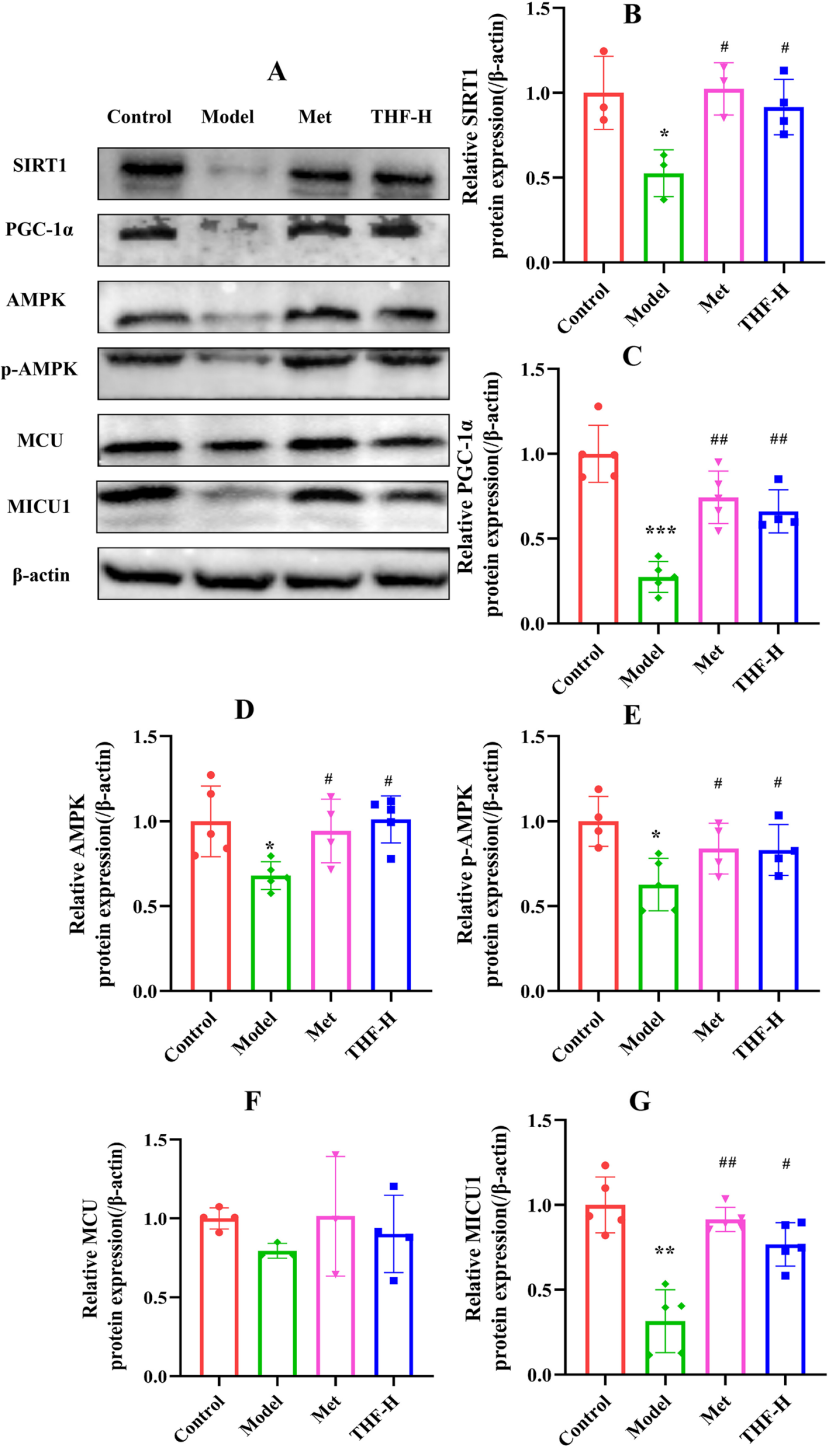
THF promoted mitochondrial energy metabolism-related protein levels in epididymal white adipose tissue of HFD/STZ-induced T2DM mice. **A**-**G** Western blot analyses of SIRT1, PGC-1α, MCU, AMPK, p-AMPK and MICU1 protein levels in Vat. β-actin was used as a loading control. Notes:^*^P < 0.05, ^**^P < 0.01, ^***^P < 0.001 *vs* Control, ^#^*P* < 0.05, ^##^*P* < 0.01, ^###^*P* < 0.001 vs Model. All the data were presented as the means ± SEMs (*n* = 3–5). Full-length blots are presented in Supplementary Figure 5

Further, the protein expression levels of AMPK, p-AMPK, SIRT1, PGC-1α, MCU and MICU1 in the Vat of each group were analyzed and found that SIRT1, PGC-1α, AMPK, p-AMPK, and MICU1 decreased in the model group, while increased after metformin or THF treatment, which were consistent with mRNA expression (Fig. 5A-G).

The above results indicated that the mRNA and protein expression levels of the mitochondrial respiratory chain, energy metabolism-related genes and the Ca^2+^ channel component protein MICU1 and MCU were reduced in E-Wat by HFD/STZ-induced, indicating that mitochondrial dysfunction and energy metabolism disorder were impaired in the E-Wat of T2DM mice, and THF improved the mitochondrial function of E-Wat in T2DM mice which might be related to AMPK-MICU1 pathway.

### THF attenuated IR via AMPK-MICU1 pathway in 3T3-L1 adipocytes

In order to further reveal that THF's improvement of mitochondrial function was related to the AMPK-MICU1 mediated _m_Ca^2+^ uptaker, we tested _m_Ca^2+^, MMP and ATP levels, and the protein expression of AMPK, p-AMPK, MCU, MICU1 in 3T3-L1 adipocyte.

Compared to the control group, the model group had significantly higher glucose levels. Simultaneously, the levels of _m_Ca^2+^, MMP, ATP and the protein expression levels of AMPK, p-AMPK, MCU and MICU1 decreased. The level of _m_Ca^2+^, MMP and ATP increased significantly after THF treatment. The results in vitro were also consistent with those in vivo.

To further investigate the relationship between AMPK and MCU, 3T3-L1 adipocytes were treated with compound C and ruthenium red, which specifically inhibited the protein expression of AMPK and MCU. The result showed the levels of mCa^2+^, MMP and ATP decreased, and the protein expression of AMPK, p-AMPK, MICU1 and MCU decreased too, resulting in elevated glucose concentration after compound C treatment. Similar results were found after intervention with ruthenium red, the difference was that there was no significant change in the protein expression of AMPK and p-AMPK, thus demonstrating that IR in 3T3-L1 adipocytes was related to the AMPK-MICU1 pathway, and AMPK was upstream of MICU1.

**Fig. 6:**
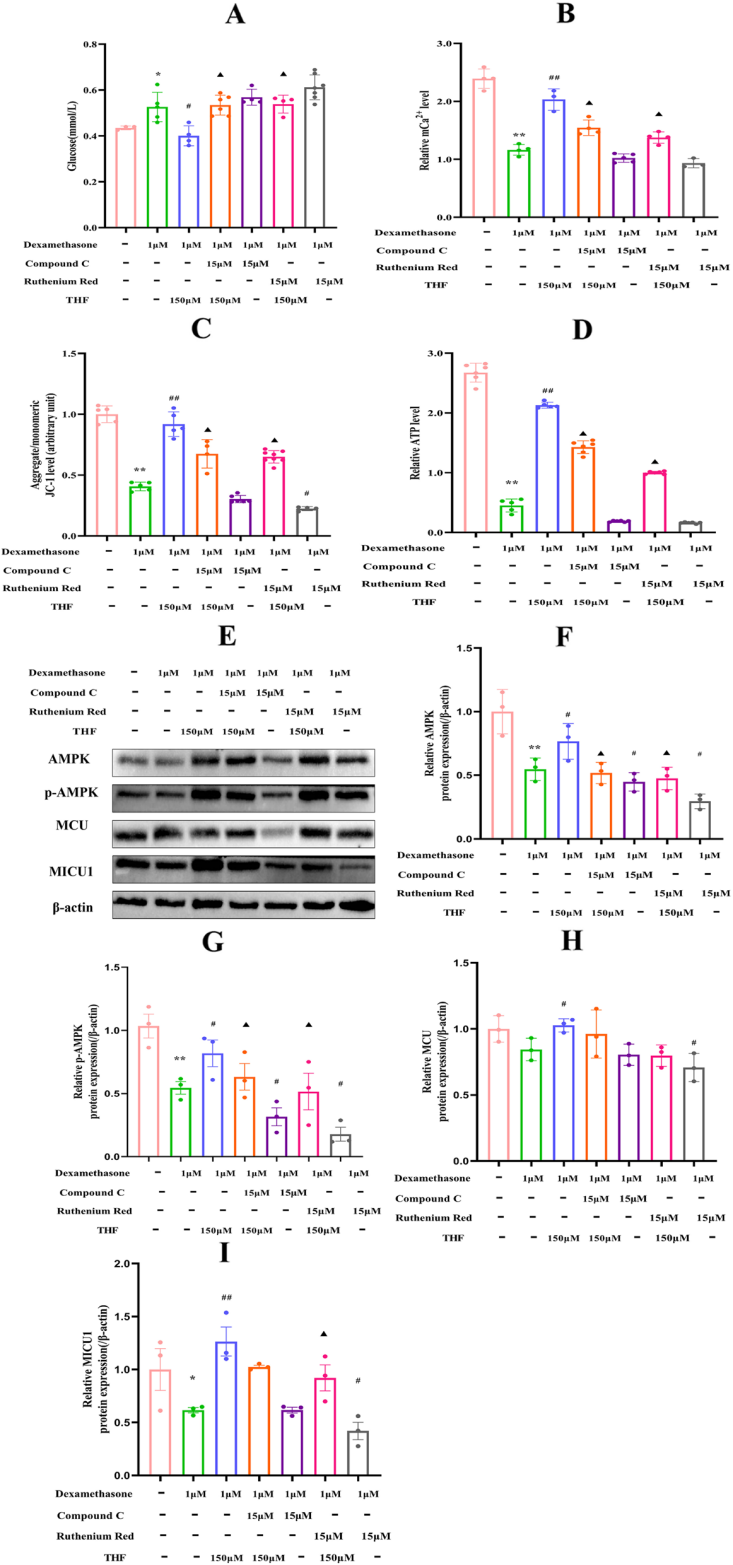
THF promoted mitochondrial function in 3T3-L1 adipocytes cells induced by dexamethasone. **A** Glucose. **B**-**D** The levels of mCa^2+^, MMP and ATP in 3T3-L1 adipocytes cells. **E**-**I** The protein expression of AMPK, p-AMPK, MICU1 and MCU. β-actin was used as a loading control. Notes:^*^P < 0.05, ^**^P < 0.01, ^***^P < 0.001 *vs* Control, ^#^*P* < 0.05, ^##^*P* < 0.01, ^###^*P* < 0.001 vs Model,^▲^*P* < 0.05, ^▲▲^*P* < 0.01, ^▲▲▲^*P* < 0.001 *vs* THF. All the data were presented as the means ± SEMs (*n* = 3–5). Full-length blots are presented in Supplementary Figure 6

The protein expressions of AMPK, p-AMPK and MICU1 were significantly decreased compared with THF group after compound C and ruthenium red treatment, which further verified THF treatment attenuated IR by regulating the expression of genes and proteins associated via the AMPK-MICU1 pathway in 3T3-L1 adipocytes (Fig. 6A-I).

## Discussion

T2DM has a high incidence and serious harm to human health, affects the metabolism of multiple organs and systems throughout the body, and causes a variety of complications [19]. IR is one of the major causes of T2DM [27], mitochondria dysfunction is tightly associated with IR, therefore, protecting mitochondrial function is a feasible approach to attenuate IR. The adipose tissue is one of the target organs for insulin action, however, how mitochondria in adipose tissue affect IR has to be further investigated.

A fundamental function of mitochondria is to produce ATP through oxidative phosphorylation and provides energy to the body. When the structure and function of mitochondria are damaged, it will directly lead to slower oxidative decomposition of glucose and reduce ATP production. Previous studies have found that under IR conditions, the mitochondrial structure is damaged and ATP production is significantly reduced [28]. After mitochondrial oxidative phosphorylation and the level of ATP production is increased, insulin sensitivity is increased and IR is further alleviated [29]. So enhancing mitochondrial function also promote insulin sensitivity, and improve insulin resistance.

Mitochondria play an essential role in the processes of energy production, signal transduction, oxidative stress and so on. Mitochondrial dysfunction is inextricably linked to metabolic diseases such as T2DM [30] through mechanisms including _m_Ca^2+^ disorder and the decreased activity of the electron transport chain complex [31]. Ca^2+^ is an important regulatory ion and mitochondrial dysfunction caused by the imbalance of _m_Ca^2+^ homeostasis is considered to be a significant pathological mechanism of diabetes [32], while MCU/MICU1 is a key molecule that regulates _m_Ca^2+^ uptake [7]. A long-term high glucose environment will cause the decrease of MCU expression, which lead to the weakening of the uptake of Ca^2+^ by the mitochondria or the imbalance of _m_Ca^2+^ homeostasis [33], and futher lead to abnormal mitochondrial electron transport chains and decrease in MMP [4, 34]. Due to the reduction of _m_Ca^2+^ uptake, the activity of rate-limiting enzymes of the tricarboxylic acid cycle is reduced, and the production of ATP is significantly reduced [35]. *Suarez J *et al*.* prove that the genes and proteins expression of MCU is related to _m_Ca^2+^ concentrations in the heart of diabetic mice [36]. The above-mentioned studies indicate that the MCU and MICU1 act as the main channel for _m_Ca^2+^ uptake and regulate the uptake of Ca^2+^ by mitochondria. Experimentally, we found similar phenomena through experiments, in the present study, the genes and proteins expression of MCU and MICU1 decreased, the uptake of _m_Ca^2+^ was reduced, MMP and ATP were also significantly reduced in the E-Wat of T2DM mice. The genes and proteins expression of MCU and MICU1 in 3T3-L1 adipocytes were also decreased under a high glucose environment. The uptake of _m_Ca^2+^ decreased, and MMP and ATP were significantly decreased too.

Ca^2+^, as the second messenger factor, is a pivotal signal in the transmission mechanism of mitochondrial energy activity. In the mitochondrial oxidative respiratory chain complexes, Ca^2+^ can enhance the activity of oxidative phosphorylation, thereby increasing the production of ATP [37]. At the same time, MCU and MICU1 are major highly selective channels for _m_Ca^2+^ uptake, and the transportation of Ca^2+^ depends on the electrochemical gradient of MMP [38]. In a high glucose environment, the MMP of 3T3-L1 adipocytes decreases, forming a vicious cycle [39]. Previous studies have shown that _m_Ca^2+^ effects membrane potential and ATP production. No research has confirmed whether decreased uptake of _m_Ca^2+^ in adipocytes in a high-glucose environment leads to abnormal MMP and ATP production. It is unclear whether AMPK participates in the regulation of _m_Ca^2+^ homeostasis in adipose tissue, thereby affecting the occurrence of diabetes. In the present study, we found systemic energy expenditure was significantly decreased, along with a significant decrease in MMP, ATP and mCa^2+^ levels in Vat of T2DM mice. The mRNA expression of COX5b, NDUFS8, SDHb, UQCRb and mitochondrial function-specific genes TFAM and COX4 decreased. The mRNA and protein expression levels of AMPK, p-AMPK, MICU1, SIRT1 and PGC-1α decreased. It could be seen that AMPK in Vat was involved in the regulation of _m_Ca^2+^, increasing _m_Ca^2+^, MMP and ATP levels.

We additionally found a decrease in protein expression of MCU and MICU1 in 3T3-L1 adipocytes cultured in a high-glucose environment. After adding AMPK inhibitor Compound C and MCU inhibitor ruthenium red, the protein expression of AMPK, p-AMPK, MCU and MICU1 decreased. It was found that the decreased expression of MCU and MICU1 in a high glucose environment evoked inhibition of _m_Ca^2+^ uptake, MMP and ATP production. Through the results, we found that the down-regulation of MCU and MICU1 mediated mitochondrial dysfunction and led to IR in 3T3-L1 adipocytes cultured in a high-glucose environment. We confirmed that the energy metabolism disorder of T2DM may be related to the mitochondrial dysfunction caused by the decreased expression of MCU and MICU1, and the abnormal _m_Ca^2+^ uptake caused by the MCU-mediated mitochondrial dysfunction, which may be the pathogenesis of T2DM.

Natural Chinese herbal medicines have been proven to have a wide range of pharmacological effects. Screening drugs that improve insulin sensitivity from natural Chinese herbal medicines may be a potential strategy for T2DM. T2DM belongs to the “Danzhuo” in TCM, which was first put forward by *Prof.* Jiao Guo from the Guangdong Pharmaceutical University. THF is composed of *Radix Notoginseng* and *Rhizoma Coptidis*, based on the theory of traditional Chinese medicine "TiaoGan QiShu HuaZuo". Studies have shown that THF has the effect of improving hepatosteatosis and glucose intolerance in diet-induced obese rats [19], but the underlying mechanism still needs to be revealed. We investigated the effect of THF on HFD/STZ-induced T2DM mice, and found that THF not only regulated glucose metabolism, but also improved insulin sensitivity. Furthermore, APNs are inflammatory cytokines associated with obesity and insulin resistance [25]. THF administration led to significant improvement in ANP level. Thus, these findings suggest that THF exerts protective effects against T2DM. Surprisingly, there were no differences in the weight of brown adipose and subcutaneous adipose among the groups, while the weight of E-Wat increased significantly in the model group and significantly adjusted by metformin or THF treatment, so we speculated that the effect of THF on improving glucose and lipid metabolism disorders may be related to E-Wat dysfunction. We futher found after THF administering, the _m_Ca^2+^, MMP and ATP increased, and the mRNA and protein expression levels of AMPK, MICU1, SIRT1, PGC1α, and MICU1 also increased in E-Wat of T2DM mice.

To investigate whether THF reduces _m_Ca^2+^ uptake via AMPK/MICU1 pathway, we then used compound C and ruthenium red, which specifically inhibited the protein expression of AMPK and MCU. The results showed that THF increased the level of _m_Ca^2+^ in 3T3-L1 adipocytes, regulated mitochondrial function, thereby improving IR, and its effect might be partly achieved by regulating _m_Ca^2+^ uptake disorders via AMPK/MICU1 pathway.

There were several limitations in the study. First, liver and skeletal muscle also play an important role in mitochondrial energy metabolism, so study the AMPK-MICU1 pathway in the liver and skeletal muscle to regulate _m_Ca^2+^ uptake disorders is also very valuable. Second, the study only investigated the relationship between AMPK and MICU1 on 3T3-L1 adipocytes, establish a tissue-specific MICU1 gene knockout model for further research may make the conclusion more convincing.

## Conclusion

In this study, we verified the effect of MICU1 as an influential regulator of _m_Ca^2+^ homeostasis in a high-glucose environment. AMPK-MICU1 might be an important pathway for adipocyte energy metabolism disorders under high a glucose environment and was involved in the pathogenesis of T2DM. Furthermore, our study showed that THF treatment attenuated diabetes by regulating adipocyte mitochondrial function by AMPK/MICU1 pathway in vivo and in vitro.

## Supplementary Information

**Additional file 1:** **Table 1.** Primers for Real-Time PCR detection.

**Additional file 2:** **Table 2.** Primary antibodies for Western blotting assay.

**Additional file 3: Supplementary Figure 5.** Effect of THF on mitochondrial energy metabolism-related protein levels in epididymal white adipose tissue of HFD/STZ-induced T2DM mice.

**Additional file 4: Supplementary Figure 6.** Effect of THF on mitochondrial energy metabolism-related protein levels in 3T3-L1 adipocytes cells induced dexamethasone.

## Data Availability

The data used and/or investigated during the present study are accessible from the corresponding author on reasonable request.

## Abbreviations

T2DM: Type 2 diabetes mellitus
THF: Tianhuang formula
AMPK: AMP-activated protein kinase
MICU1: Mitochondrial Ca^2+^ uptake 1
mCa^2^^+^: Mitochondrial calcium
HFD: High fat diet
STZ: Streptozocin
FBG: Fasting blood glucose
VAT: Visceral adipose tissue
DXM: Dexamethasone
MMP: Mitochondrial membrane potential
p-AMPK: Phosphorylated AMPK
SIRT1: Sirtuin1
PGC-1α: Peroxisome proliferator-activated receptor-γ coactivator-1α
MCU: Mitochondrial Ca^2+^ uniporte
DM: Diabetes mellitus
IR: Insulin resistance
OXPHOS: Oxidative phosphorylation
ATP: Adenosine triphosphate
THF-H: High-dose THF
THF-L: Low-dose THF
MET: Metformin
OGTT: Oral glucose tolerance test
AUC: Area under the curve
LDL-C: Low density lipoprotein cholesterol
TC: Total cholesterol
TG: Triglycerides
HDL-C: High density lipoprotein cholesterol
NEFA: Nonestesterified fatty acid
APN: Adiponectin
MMP: Mitochondrial membrane potential
BAT: Brown adipocytes
VAT: Visceral adipose tissue
