# Supplementary material for: Tianhuang formula regulates adipocyte mitochondrial function by AMPK/MICU1 pathway in HFD/STZ-induced T2DM mice
Source: BMC Complement Med Ther. 2023 Jun 19;23:202. doi: 10.1186/s12906-023-04009-5 (PMC10278277; doi:10.1186/s12906-023-04009-5)
Supplement: Supplementary file 1 — Additional file 1: Table 1. Primers for Real-Time PCR detection. [file 12906_2023_4009_MOESM1_ESM.docx]

**Table. 1 Primers for Real-Time PCR detection.**

| Gene | Sequence 5’→3’ |
| --- | --- |
| Mouse *AMPK* | F：ATGATGAGGTGGTGGAGCAGAGG  R：AGTGAGAGAGCCAGAAAGGGAGTG |
| Mouse *AMPKα1* | F：AACCTGAGAACGTCCTGCTTGATG  R：TGACTTCTGGTGCGGCATAATTGG |
| Mouse *MICU1* | F：ACAACAGTCCTCTCCACTCCTCAG  R：CCTCCATGTCTACCTCTCCGTCTC |
| Mouse *MCU* | F：TGCAAGAGGAGGATCGGGGAATC  R：AAGTCATCGAGGAGCAGGAGGTC |
| Mouse *Actin* | F：CTACCTCATGAAGATCCTGACC  R：CACAGCTTCTCTTTGATGTCAC |
| Mouse *Nrf1* | F：ATCTGGCTGCTGCAGGTC  R：GGCTCTGAGTTTCCGAAGCA |
| Mouse *SIRT1* | F：TCGGCTACCGAGGTCCATA  R：AACAATCTGCCACAGCGTCA |
| Mouse *COX4* | F：CCCAAACCAGGTGGCAGAAAA  R：ATCTTCACAGTAGTCGTGGTGTGG |
| Mouse *UQCRb* | F：CGCGTCTATCTTCTGTCCCA  R：CCACTACAAACGGCGGC |
| Mouse *TFAM* | F：GCAGCAGGCACTACAGCGATAC  R：TTCCCATTCCCTTCCCAGACTGAG |
| Mouse *SDHb* | F：CTCAGGAAGGCAAGCAGCAGTATC  R：ATTTGTCTCCGTTCCACCAGTAGC |
| Mouse *COX5b* | F：CATGGCATCTGGAGGTGGTGTTC  R：CTGGTGCCTGAAGCTCCCTTTG |
| Mouse *PGC-1α* | F：TCGTCATCCACCTCCTCGTTCTC  R：TGCTGCTGCTGCTGCTGTTG |
| Mouse *NDUFS8* | F：AGACCCATGAGGAGCTGCTGTAC  R：CTGGATGTTGGCGGCGATCTC |
