# Supplementary material for: Tianhuang formula regulates adipocyte mitochondrial function by AMPK/MICU1 pathway in HFD/STZ-induced T2DM mice
Source: BMC Complement Med Ther. 2023 Jun 19;23:202. doi: 10.1186/s12906-023-04009-5 (PMC10278277; doi:10.1186/s12906-023-04009-5)
Supplement: Supplementary file 2 — Additional file 2: Table 2. Primary antibodies for Western blotting assay. [file 12906_2023_4009_MOESM2_ESM.docx]

Table . 2 Primary antibodies for Western blotting assay.

| Antibody | Cat No. | Manufacturer | Species |
| --- | --- | --- | --- |
| SIRT1 | PA5-17074 | CST | Rabbit |
| PGC-1α | ab106814 | Abcam | Rabbit |
| AMPK | ab32047 | Abcam | Rabbit |
| Phosoho-AMPKα | ab131357 | Abcam | Rabbit |
| MICU1 | ab224161 | Abcam | Rabbit |
| MCU | ab219827 | Abcam | Mouse |
| β-ACTIN | ab8227 | Abcam | Rabbit |
