# Supplementary material for: Tianhuang formula regulates adipocyte mitochondrial function by AMPK/MICU1 pathway in HFD/STZ-induced T2DM mice
Source: BMC Complement Med Ther. 2023 Jun 19;23:202. doi: 10.1186/s12906-023-04009-5 (PMC10278277; doi:10.1186/s12906-023-04009-5)
Supplement: Supplementary file 3 — Additional file 3: Supplementary Figure 5. Effect of THF on mitochondrial energy metabolism-related protein levels in epididymal white adipose tissue of HFD/STZ-induced T2DM mice. [file 12906_2023_4009_MOESM3_ESM.docx]

Supplementary Information

Fig 5: The red box position indicates the cutting position

|  | Control Model Met THF-H |
| --- | --- |
| SIRT1 | 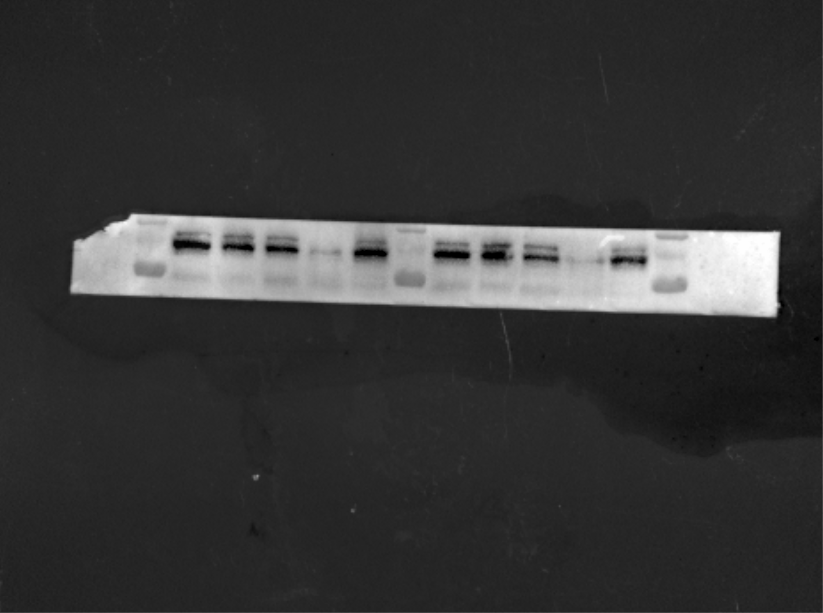 |
| PGC-1α | 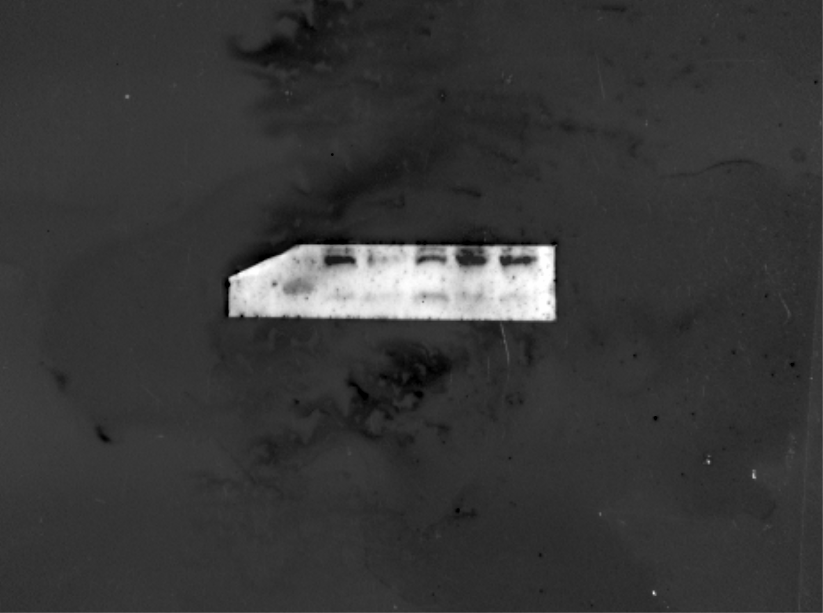 |
| AMPK | 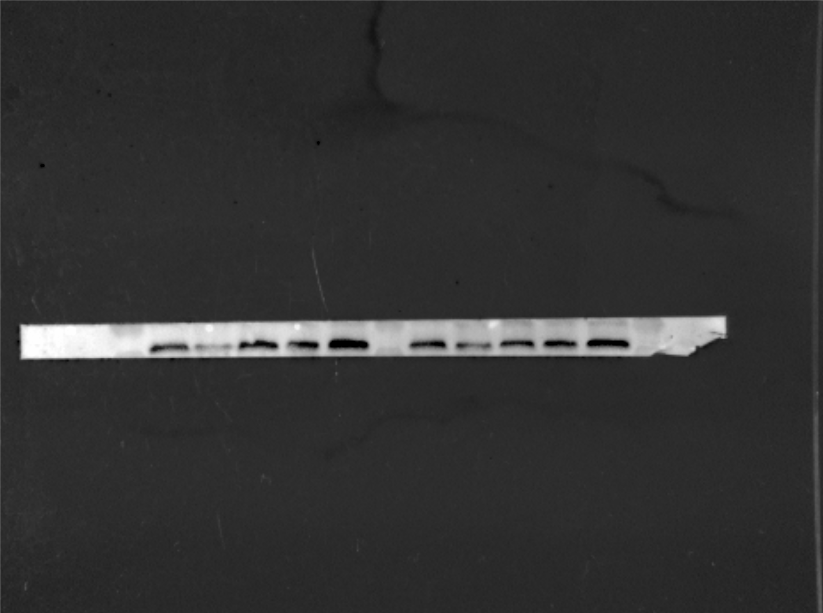 |
| p-AMPK | 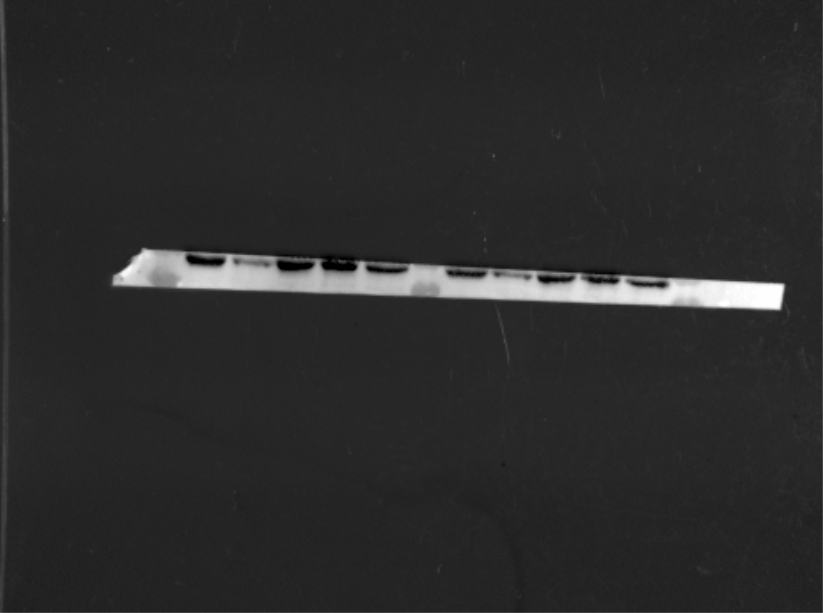 |
| MCU | 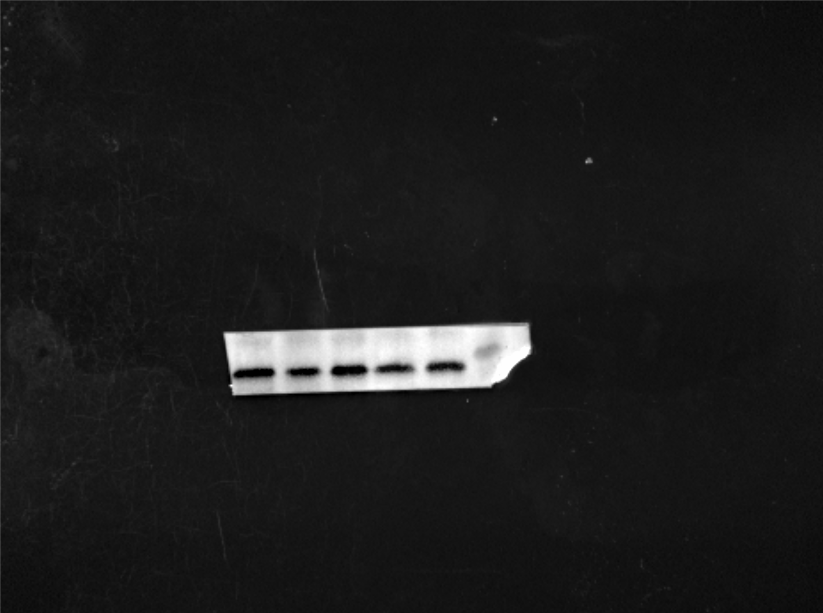 |
| MICU1 | 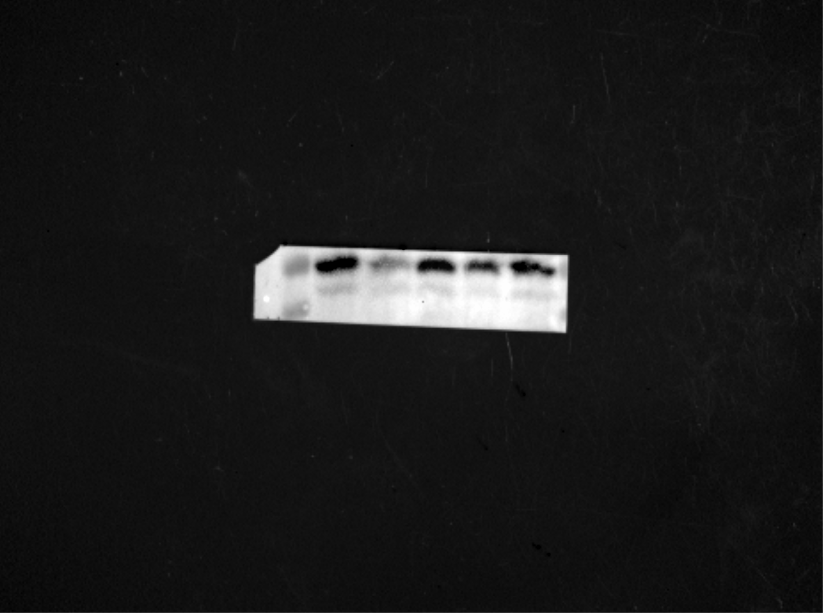 |
| β-actin | 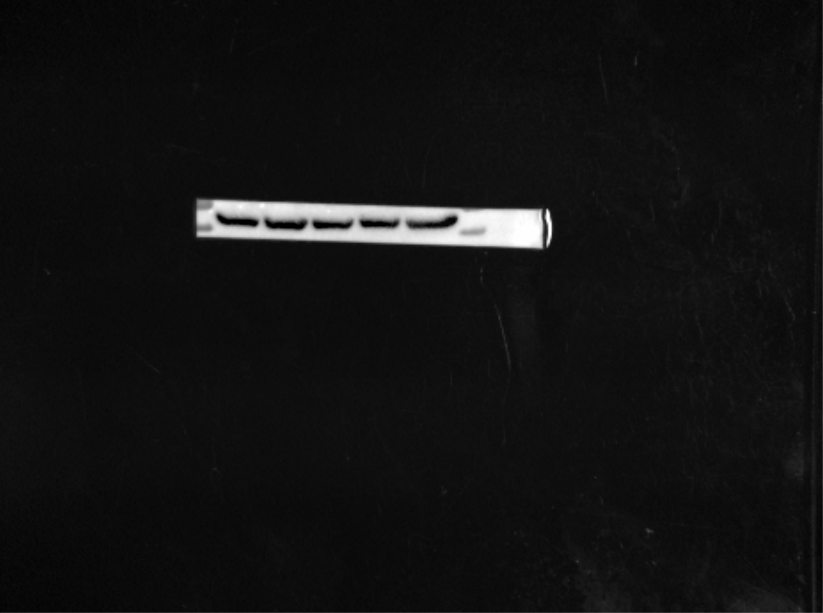 |
